# The "Dry Treatment" of Suppurative Inflammation of the Middle Ear

**Published:** 1884-03

**Authors:** A. W. Calhoun

**Affiliations:** Professor of Eye, Ear and Throat Diseases in the Atlanta Medical College, Atlanta, Ga.


### THE "DRY TREATMENT" OF SUPPURATIVE INFLAMMATION OF THE MIDDLE EAR.*

BY A. W. CALHOUN, M.D.,

*Professor of Eye, Ear and Throat Diseases in the Atlanta Medical College, Atlanta, Ga.*

It is a very common custom for physicians to direct in the above disease that the ear be "syringed several times daily till the discharge ceases." The fact that absolute cleanliness is a *sine qua non* in the cure of this affection naturally leads to the conclusion that water must be used much and often. For a long time I held to this view myself, until actual observation and experience suggested to me that water could be used to an extent that hindered rather than aided a cure, and taught me a plan of treatment most satisfactory to myself and beneficial to my patients.

While the syringe has been too much and too often used, it cannot be dispensed with altogether, for in its place it is a very valuable adjunct in the treatment of discharges from the middle ear. But in the hands of the ordinary nurse or attendant, the water is improperly thrown into and down the external auditory canal, simply because of a want of anatomical knowledge of the parts, and because of the fear of giving pain to the patient and doing damage to the ear.

A chronic discharge from an ear, presupposes the existence of a perforation of the drum membrane and consequent exposure of the delicate mucous membrane lining the cavity of the tympanum, all of which has been produced by an acute inflammation at some previous time.

Now, the too frequent use of water keeps the exposed tympanic mucous membrane constantly moistened, which renders the ear quite susceptible to recurrent attacks of acute, painful, catarrhal inflammations, commonly called "risings in the head." It is a well known fact that any exposed mucous membrane elsewhere in the body, when kept more or less continually moistened, will suppurate much more profusely that when dry. This is eminently so of the mucous membrane of the middle ear, and it would appear that some plan should have been known long since, of thoroughly cleansing the ear without too frequently repeated syringing. I do not agree

*This article first appeared in the Transactions of the Medical Association of Georgia, but has been re-written with some important changes for the JOURNAL.

with him who says "the syringe should not be used at all," for without it, it is next to impossible to observe perfect cleanliness, especially in an ear with a long continued and profuse discharge. Besides, it is necessary to use the syringe to rid the ear of the "caked" powders which may have been used in the treatment of the disease.

The plan I have found most serviceable is as follows: The patient must supply himself with a good ear syringe (one ounce gutta percha syringe with a bulb nozzle) and a quantity of absorbent cotton, and the attendant must be taught the proper use of both. For the first few days the ear must be syringed with warm salt water twice daily, and mopped to thorough dryness immediately afterwards by means of the absorbent cotton twisted upon and overlapping the end of a match. This close attention to cleanliness usually diminishes the discharge in a few days, when the ear should be syringed *only once daily*, but the absorbent cotton should now be used every few hours, so as not to allow any of the constantly forming pus to remain in the external auditory canal at all. The patient himself, if not too young, can be taught to use the cotton, when he can carry some in his pocket and mop the ear almost every hour in the day. At this point in the treatment, the use of astringent and disinfecting powders comes best into play. I usually prescribe the pulv. boracic acid (unless MINUTELY PULVERIZED it is worse than useless), and pulv. alum with just enough of the powdered lycopodium in it to keep it dry. An intelligent nurse can be easily taught the use of these, otherwise it is best for the physician himself to apply them.

Syringe the ear, for instance, each morning with warm salt water, and mop it with the absorbent cotton till the cotton comes out free from moisture. Then turn the head upon the side so as to have the auditory canal looking directly upward, insert the speculum and pour in the powder, and gently, but firmly, pack it to the bottom of the canal with a large knitting needle, or probe of any kind, and hold the powder in place by means of a little cotton. If towards noon or night the cotton becomes wet with the discharges, the ear should be again well dried with the absorbent cotton and refilled with powder, repeating the same thing each day, and several times daily if necessary. It is well to alternate with the two powders above mentioned, using each two or three days, because the ear soon gets accustomed to the one and its good effect is somewhat lost; moreover, it is said that the too long use of the alum powder will produce furuncular inflammation in the auditory canal.

Few ears will resist the persistent use of this treatment, but now and then a case will present itself which requires some modification in its management. In such cases I combine with the above treatment that of some astringent fluids. In a case which had resisted the careful and vigorous treatment with the powders, etc., I would give the following directions to my patient: On retiring at night, mop the ear well, then pack it full of the powder. On the following morning, syringe it, if necessary, and use the absorbent cotton, then fill the canal with warm tincture of myrrh and glycerine (equal parts), combined with sulphate of zinc; or substitute lysterine for the tincture of myrrh and use in the same way. At noon do the same thing, and at night repeat the powder. In the meantime the patient must be constantly drying his ear by means of the absorbent cotton.

Almost from the moment the powders are used, the offensive odor from the most fetid discharge ceases, and one of the most disgusting features in the case is happily disposed of.

While the partially "dry treatment" is most excellent, I would advise against any one becoming so wedded to it as to lose sight of the fact that certain cases will require the more frequent use of the syringe than others, and again, to discard the syringe is to throw aside the means of *absolute cleanliness*, without which a cure, by whatever mode of treatment (except mere accident), is simply out of the question.

